# Three-dimensional hierarchical porous carbon structure derived from pinecone as a potential catalyst support in catalytic remediation of antibiotics†

**DOI:** 10.1039/c9ra10638c

**Published:** 2020-02-28

**Authors:** S. O. Sanni, E. L. Viljoen, A. E. Ofomaja

**Affiliations:** Biosorption and Wastewater Treatment Research Laboratory, Department of Chemistry, Faculty of Applied and Computer Sciences, Vaal University of Technology P. Bag X021 Vanderbijlpark-1900 South Africa aus_ofomaja@yahoo.com augustineo@vut.ac.za

## Abstract

In this study, pinecone was converted *via* two stage pyrolysis to produce low cost activated carbon. Furnace pyrolysis was used in the first step to convert pinecone to carbonized material, followed by microwave pyrolysis of the carbonized material activated with KOH to obtain activated carbon (ACK) materials as a suitable catalyst support. The ACK samples were characterized by their morphology, structural, adsorption and electrochemical properties. The optimized ACK 2.24-16 prepared from the pinecone had a complex three-dimensional (3D)-hierarchical porous structure, with an abundance of micropores and mesopores compared to other ACK samples judging from the high iodine number (1900 mg g^−1^) and the methylene blue number (4000 mg g^−1^) capacity. The optimized ACK 2.24-16 had the highest current response and least charge transfer resistance, along with moderate surface area (427 m^2^ g^−1^) as a promising photocatalyst support. The 3D hierarchical porous ACK significantly assisted catalyst dispersion, and enhanced visible light absorption and fast interfacial charge transfer. This work shows the promising aspect of utilizing pinecone to produce a low-cost photocatalyst support for environmental remediation.

## Abstract 2

Three-dimensional hierarchical porous carbon prepared from microwave pyrolysis of pinecone. The carbon material aid Ag–AgBr photocatalytic attributes and enhanced the activity on removal of tetracycline.

## Graphical abstract

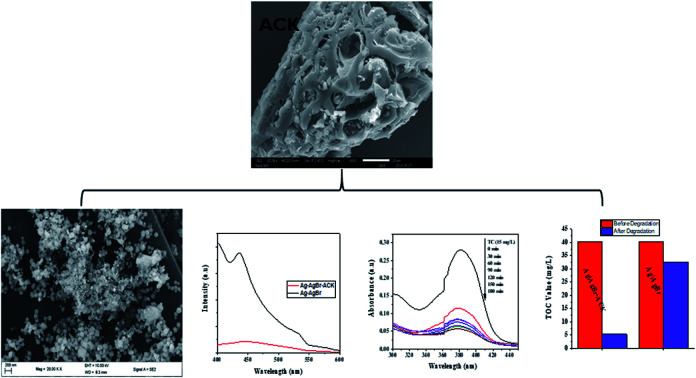

## 1. Introduction

The global environmental demand for clean water is increasing, especially with the detection of emerging contaminants like pharmaceutical and personal care products (PPCPs) in water bodies. These PPCPs at lower concentration levels have serious adverse and relentless effects on ecosystems and human health,^1,2^ therefore their elimination is required to reduce their adverse impacts on the environment. Heterogeneous photocatalysis utilizing metal semiconductor materials as catalysts under light exposure at relatively low temperature, has received considerable attention for the efficient removal of these PPCPs.^3^ However, the application of these photocatalysts for enhanced performance is hindered by aggregation and agglomeration of the catalyst materials during preparation, weak light harvesting efficiency, inefficient separation and fast recombination rate of photoexcited charge carriers, and poor recovery from solution after degradation activities.^4–6^ The immobilization of the catalytic active phase on high surface area insoluble materials as a cocatalyst in the degradation process is a promising approach in obtaining highly efficient and stable photocatalytic composites.^7–12^

The higher specific surface area, excellent electron conductivity, active binding sites for catalyst dispersion and their relative chemical inertness are exceptional attributes for utilizing carbonaceous materials (graphene, graphene oxide, carbon nanotube and activated carbon) to support photocatalysts compared as support to other materials.^13–15^ Low cost of production, abundance, inexpensive matrix with nontoxicity are key factors towards selection of activated carbon (AC) among these carbonaceous materials for catalyst dispersion.^16,17^ In the optimization of activated carbon (AC) as a catalyst support, the desire should not only be based on a high specific surface area for effective adsorption–desorption of solution and abundant functional groups for catalyst dispersion judging from previous works.^18–20^ The AC must also have exceptional interconnected porous structure channel with accessible pore volume, favorable for light harvesting, aid electrons transfer after generation and assist separation of photo-generated charge carriers, high adsorption and diffusion of targeted pollutants.^21–23^

The AC porous channels in form of 3D hierarchical network structures have shown promising attributes in fuel cells, supercapacitors and environmental remediation.^24–27^ However, previous works devoted in development of these 3D hierarchical porous AC structures relies on utilization of high-price template, requirement of energy intensive routes, and the usage of corrosive chemicals, which pose huge concerns on environmental sustainability and production costs.^12,20^ A more sustainable approach to develop 3D hierarchical porous AC structure exploring sustainable resources like biomass is crucial to alleviate the preparation cost, environmental impact and also enhance the value of the biomass material.^28,29^ Previous works have explored agricultural biomasses, waste residues and wood as renewable precursors for generating 3D hierarchical porous AC structure materials as catalyst support for pollutant removal with high activity.^12,30,31^ The unique 3D hierarchical porous AC structure potential as a catalyst support depends strongly on the type of agricultural biomass precursor, the activation methods and the heating method during carbonization.

Particularly, pinecone biomass is abundantly available throughout the world, a valuable product in waste remediation^32^ due to its excellent chemical composition.^33^ The ovulate pinecone are rich in cellulose, hemicellulose, lignin, resin and tannins,^34^ that serve as the biomass source for AC synthesis. Host of carbon materials from pinecone have shown good performances in many fields such as supercapacitors, energy production, adsorbent and electrocatalysts.^35–38^ Activated carbon from various agricultural sources have been used as catalyst supports.^31,39^ Regarding activation condition, AC produced *via* mixing of the precursor with chemical activating agent especially potassium hydroxide (KOH), has shown potential for good formation of 3D hierarchical network structure with large surface area.^40^ This emanates from interaction of potassium atom with the carbon structure of the AC by means of dehydration and degradation.^41,42^ Microwave-assisted pyrolysis instead of conventional oven heating in terms of heating condition has shown promising attributes for generation of 3D AC interconnected porous channels. This may be ascribed to the microwave exceptional fast, efficient and selective heating mechanism.^43^ There is no reports in literature regarding the optimization of different preparation parameters on activated carbon produced from pinecone, as a catalyst support in heterogeneous catalysis for antibiotics remediation.

Inspired by these facts, the focus of this study was to construct 3D hierarchical porous AC structure through microwave pyrolysis of KOH impregnated pinecone as a potential photocatalyst support (ACK). The characteristic properties of 3D hierarchical porous AC structures with abundant oxygenated functional groups were established, along with their efficient adsorption attributes is discussed in this study. Also in this work, the contribution of optimized ACK in the silver–silver bromide carbon composite (Ag–AgBr ACK) as catalyst support, prepared *via* thermal polyol route was investigated. Furthermore, the practical application of as-prepared nanocomposite was carried out on the catalytic reduction of tetracycline antibiotic under visible light irradiation.

## 2. Materials and method

### 2.1. Materials

Agricultural biomass pinecones were collected from Vaal University parking space, South Africa and will serve as the precursor for carbon production. The pinecones were washed to remove impurities such as sand and leaves from the material. The washed cone was dried in a conventional oven at 90 °C for 48 h. The scales on the cones were removed and crushed using a pulveriser to make a fine powder. All the reagents were used as received and the water used in all these experiments was purified with a Millipore system.

### 2.2. Activated carbon preparation

This work adopts the established two-stage pyrolysis method for preparing AC.^44^ The pinecone powder (approximately 15 g) weighed on an aluminum foil, loaded into a quartz reactor and then further placed in a tubular furnace (Carbolite Gero, MTF 12/38/250). Prior to carbonization of the powder, nitrogen gas passed through the furnace to eliminate air from the quartz reactor for about 10 minutes before heating up the furnace. The material was heated using a heating rate of 10 °C min^−1^ and carbonized at 600 °C for 2 h under inert atmosphere of nitrogen flow (50 mL min^−1^) to obtain the carbonized material (CM). The CM was allowed to cooled, washed repeatedly with distilled water and dried overnight at 80 °C. A yield of about 40 wt% was obtained for the carbonized material, which was further subjected to chemical activation for conversion into activated carbon. The chemical activation of the BCR (5 g) with a particle size of less than 300 μm were carried out with a 25 mL KOH (99%, Merck) solution, at different mass ratio of KOH to carbonized material or impregnation ratio (IR) (0.56–3.36). The prepared mixtures were impregnated for 24 h and the solution was later dried in oven at 80 °C for 12 h to remove any remaining moisture present in the material. The dried sample placed in Duran bottle and flushed with Nitrogen (50 mL min^−1^) for 30 min, then pyrolyzed in the microwave reactor (model LG MH8042GM, maximum power of 1000 W at a frequency of 2450 MHz) at a constant power of 400 W for different times (8–24 min) with 4 minutes difference interval. The produced activated carbon samples were cooled to room temperature, washed with 0.1 M hydrochloric acid (99%, Sigma Aldrich) and hot distilled water to remove any impurities until the pH is between 6 and 7. The ACs were further dried at 105 °C overnight, and named as ACK-*a-b*, where *a* represents mass ratio of KOH to carbonized material (impregnation ratio) and *b* is the microwave pyrolysis time during the microwave process. The detailed procedure of the fabrication of photocatalyst composites such as silver–silver bromide activated carbon (Ag/AgBr-ACK), silver activated carbon (Ag-ACK), silver bromide activated carbon (AgBr-ACK) and silver–silver bromide (Ag/AgBr) can be found in the ESI.† The photocatalytic activities of these prepared materials on degradation of tetracycline antibiotics are also presented in ESI.†

### 2.3. Characterization

The percentage yield (*Y*%) was calculated as the ratio as the ratio of the dry weight of produced pine cone-activated carbon (*W*_ACK_) to weight of the carbonized material (*W*_CM_)^45,46^ as depicted below:1
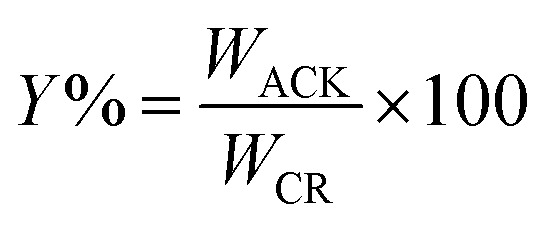


The morphology of CM, ACKs and Ag/AgBr-ACK samples were determined by scanning electron microscopy (Zeiss Leo 1430 VP). The ACKs functional groups were investigated by Fourier transform infrared spectrometer (PerkinElmer spectrum 400) within the range of 600–4000 cm^−1^. X-ray diffraction (XRD) patterns were obtained by using Bruker diffractometer AXS with CuKα radiation source (source light at wavelength of 0.1541 nm) with scan range of 10 to 80°. Thermal analysis on each sample was carried out using PerkinElmer STA 6000 thermal simultaneous analyzer at heating rate of 5 °C min^−1^ from 30 to 900 °C under nitrogen purge stream of 20 mL min^−1^. The BET surface area, average pore size distribution is estimated by N_2_ adsorption at 77 K using a Micromeritics (Australia) Tristar 3000 analyzer coupled to a VacPrep 061 degassing unit.

The point of zero charge (pH-PZC) was determined using 6 points experiment. The ACK sample (100 mg) along with 50 mL of 0.1 M potassium nitrate (KNO_3_) solution was agitated at 25 °C for 48 h to allow it to reach the equilibrium state.^47^ The initial pH of KNO_3_ solution (2, 4, 6, 8, 10 and 12) was adjusted using 0.1 M HCl acid and 0.1 M NaOH. After shaking for 48 h, the final pH was measured using Hach pH meter. The electrochemical properties of prepared ACK samples were conducted in a standard three-electrode workstation (Biologic SP 240 potentiostat). A glassy carbon electrode (GCE, 5 mm in diameter) was utilized as the working electrode, while platinum wire and Ag/AgCl (in saturated KCl) were used as the counter electrode and a reference electrode respectively. The ACKs samples suspension were prepared by dispersing 5 mg of material into 0.5 mL DMF solution followed by ultrasonication for 1 h. A volume of 20 μL of the ACK suspension was dropped onto the surface of GCE by using a micropipettor and then dried at room temperature. The cyclic voltammetry (CV) method was performed in 6 M KOH solution at a scan rate of 50 mV s^−1^ from −0.8 V to +0.2 V. The electrochemical impedance spectroscopy (EIS) was tested between frequency ranges of 100 kHz to 10 mHz with a perturbation amplitude of 5 mV.

### 2.4. Methylene blue and iodine adsorption capacity for ACK

The methylene blue adsorption capacity also called methylene blue number (MBN) is defined as the maximum amount of dye adsorbed on 1.0 g of adsorbent. The MBN is a measure of mesopores content (2–5 nm) present in produced ACK samples. The batch adsorption of MB (99%, Sigma Aldrich) was conducted in a set of 100 mL plastic bottles containing 0.01 g ACK along with 40 mL dye solution at various initial concentrations (10, 25, 50, 100, 250, 500, and 1000 mg L^−1^) at pH of 12. The plastic bottles were agitated in a shaker at room temperature with a shaking speed of 120 rpm until the equilibrium was reached after 2 h. The samples were filtered and the residual concentration of MB in the supernatant solution was analyzed using a double beam UV-Vis spectrophotometer at 665 nm. The amount of MB uptake per unit mass of adsorbent at equilibrium, *Q*_e_ (mg g^−1^), was calculated by:2
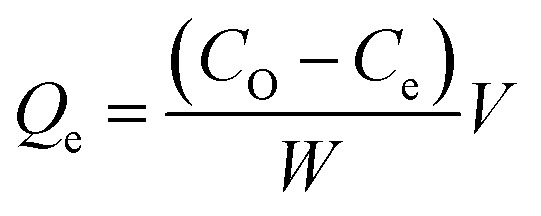
where *C*_o_ and *C*_e_ (mg L^−1^) are the liquid-phase concentrations of MB dye at initial and equilibrium, respectively. *V* (L) is the volume of the solution, and *W* (g) is the mass of ACK used. The initial pH of the dye solution was adjusted by addition of 0.1 M of HCl or NaOH.

The iodine adsorption capacity represented as iodine number (IN) is a good measure of microporosity of the AC (up to 2 nm). The IN indicates milligrams of iodine adsorbed by a gram of activated carbon (mg g^−1^), is determined by using the ASTM D4607-94 method for characterizing ACK samples. A mass of 0.1 g ACK samples were placed in a dry 100 mL volumetric flask, then fully wetted with 10 mL of HCl (5 wt%). The mixture was boiled on the hot plate for about 30 s and allowed to cool. After cooling down to room temperature, 100 mL of iodine (Sigma Aldrich) standard solution (0.1 mol L^−1^) was added, and then shake for 5 min. The mixture was filtered, then 50 mL of the filtrate were transferred with a pipette into a 250 mL volumetric flask, and further titrated with sodium thiosulfate (0.1 mol L^−1^) until the solution became pale yellow. A volume of 2 mL of starch indicator solution (5 g L^−1^) were added and the titration was continued with sodium thiosulfate until the solution became colorless.^47^

## 3. Results and discussion

### 3.1. Yield and characteristics properties of carbon samples

The high carbon and low ash content of pine cone (PC) biomass^48^ makes the pine cone an ideal precursor for the production of activated carbon. The influence of impregnation ratio (IR) of the chemical activating agent and the microwave pyrolysis time (MPT) plays a major role in the final yield of the produced activated carbon. The chemical activating agent (KOH) acts as dehydrating agents, whereby they penetrate deep into the carbonized material structure,^49,50^ which results in decomposition of the large organic molecules present in the biomass to smaller molecules.

**Fig. 1:**
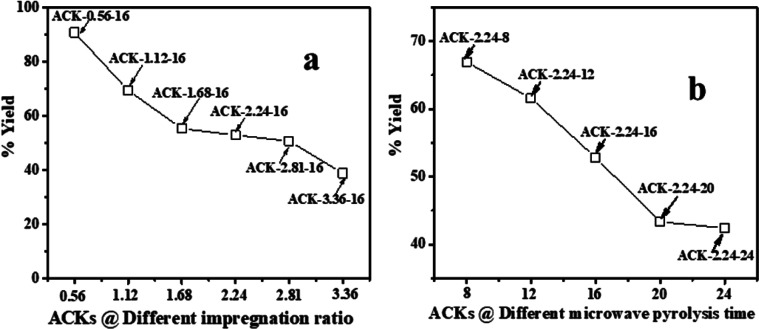
% yield of ACK at different (a) impregnation ratio and (b) microwave pyrolysis time.

The ACKs yield decreases from 91 to 39% as the IR increases from 0.56 to 3.36 (Fig. 1a), while an increase in MPT from 8 to 24 min at IR of 2.24 results in decrease in yield from 66 to 41% (Fig. 1b). The yield of ACK reduces with increasing IR and MPT, as large amount of carbon is burn-off, though the yield of ACK shows no significant changes when IR is 1.68 to 3.36. The yield variation of ACK from MPT at 8 to 24 min is significant, highlighting MPT as variable is more sensitive to IR in obtaining high yield of ACK. High ACK yield is obtained at low IR and MPT, which is ascribed to weak elimination of volatiles from the BCR due to poor activation from activating agent and pyrolysis time.^51^ Herein at higher IR and microwave time, decomposition of cellulose, hemicellulose and lignin in BCR results to producing more volatiles, which explains lower yield of ACK. The eliminated volatile matters paves access for good porous structure formation from inaccessible channel, as intense gasification of surface carbon atoms occurs with fast weight loss with increasing IR and MPT.^51,52^

**Fig. 2:**
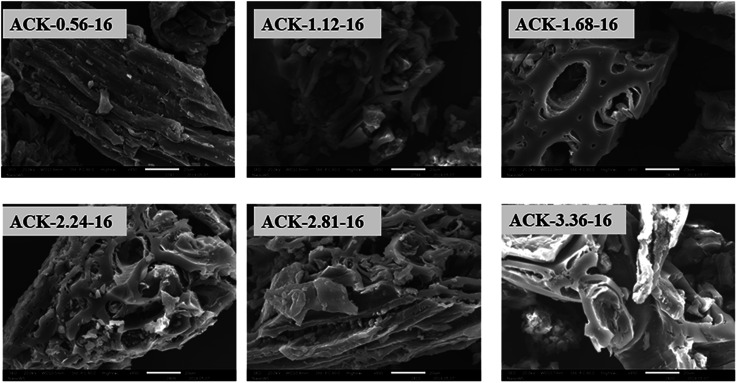
SEM images of ACKs at different impregnation ratio.

**Fig. 3:**
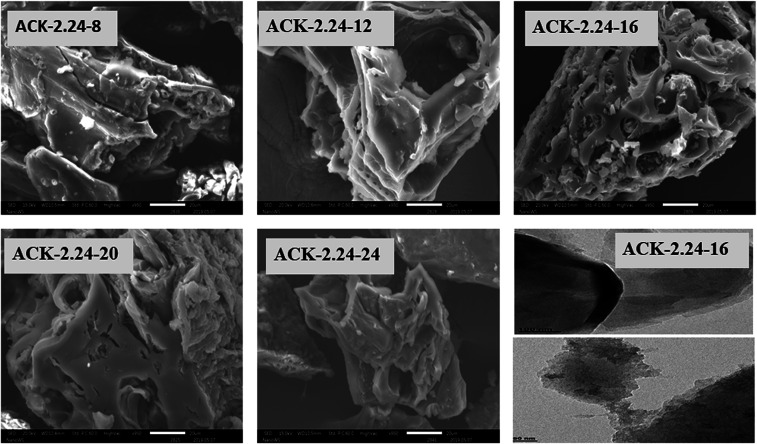
SEM images of ACKs at different microwave pyrolysis time and TEM image of optimized ACK sample.

The surface morphology of produced ACKs at different IR and MPT are further shown in Fig. 2 and 3. Carbonized material image as shown in Fig. S1† has small open channel, however upon impregnation with KOH and subjected to microwave activation, the porous structural channel becomes pronounced in produced ACKs samples in Fig. 2. During microwave activation, volatile matters are released along with formation of small rudimentary structure, as they are less developed at lower IR (0.56–1.12) in Fig. 2, which could adversely affect their adsorption and catalytic properties. The porous channel in form of three-dimensional (3D) hierarchical network becomes evident at IR of 1.68–2.81, which is due to elimination of excess volatile matters during the activation process.^41,53^ Further increment in IR to 3.36, the 3D hierarchical porous framework becomes clogged because of over gasification of pinecone and this related to the low weight loss for this ACK sample from Fig. 1a. The 3D hierarchical porous framework is well developed with ACK-2.2.4-16 sample, as well will favours high IN and MBN adsorption capacity (as further discussed in adsorption experiment). The 3D hierarchical porous framework of the ACK is also crucial for boosting the photocatalytic reaction because of the abundance of porous channels that will aid photocatalyst uniform dispersion without agglomeration.^54^ The developed 3D hierarchical porous framework would also promote light harvesting of photocatalyst material, interfacial charge carrier mobility and facilitate reactant transport into the catalyst's inner surface.^55,56^

From Fig. 3, the 3D hierarchical porous structure of ACK at low MPT (ACK-2.24-8) are not developed, depicting incomplete formation of ACK sample. Herein, the high yield of ACK-2.24-8 (Fig. 1b) shows that the volatile matters present in the pinecone were not fully eliminated for good formation of porous structure. However, the 3D hierarchical porous structure becomes pronounced along with a reduction in the ACK yield, upon an increment in MPT from 12–20 min, as surface impurities are eliminated from the surface of ACK. However, an augmentation in microwave time to 24 min with low yield (Fig. 1b), causes the existing channel to be blocked or destroyed due to over gasification of pinecone which is unfavorable for adsorption and catalytic properties. Hence, it can be inferred that KOH activation with microwave pyrolysis aided in better development of 3D hierarchical porous structural for ACK samples. The TEM micrograph of ACK-2.24-16 sample is presented in Fig. 3, with a well-developed porous structure that are attributed to presence to micropores and mesopores resulting from the KOH chemical activation.

**Fig. 4:**
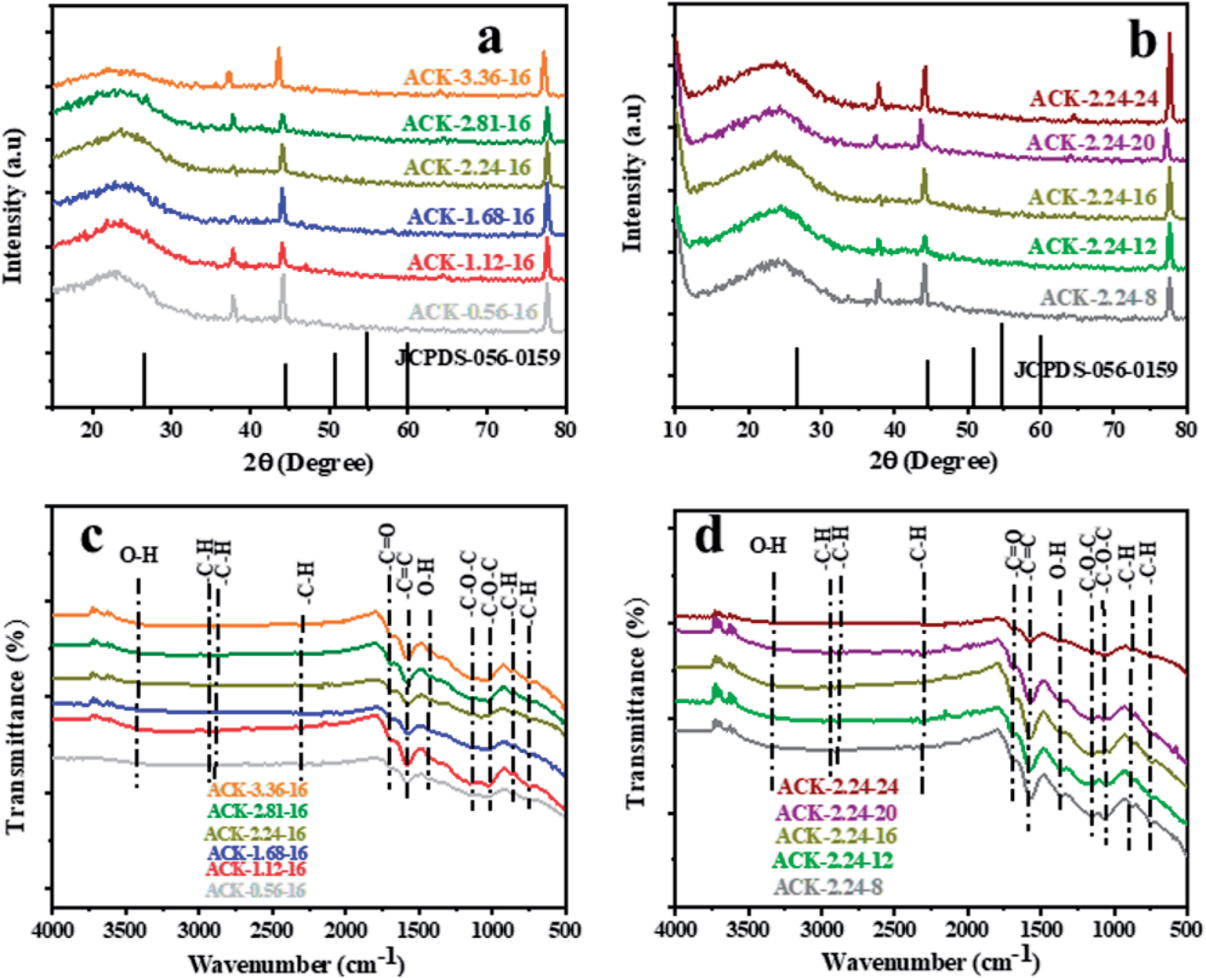
X-ray diffraction pattern and FTIR spectra of ACKs at different (a and c) impregnation ratio and (b and d) microwave pyrolysis time.

The XRD pattern of ACK prepared at different IR and MPT are depicted in Fig. 4a and b. A broaden peak is observed between range of 20–30, while a sharp peak at 43.5 is also prominent and these two peaks are ascribed to the 002 and 100 diffraction planes of carbon pattern. Herein, the prepared ACKs at 002 lattice plane are indication of amorphous carbon with carbon rings that are disorderly stack up.^57^ The ACKs at 100 plane are composed of turbostatic structure with minute presence of graphite like microcrystallites^57,58^ which is good for electrical conductivity of ACK.^59^

The FTIR spectra of ACKs at different IR and MPT are shown in Fig. 4c and d, respectively. The broad peaks around 3420 cm^−1^ is ascribed to stretching vibrations of OH bonds from the water molecules adsorbed on the surface of prepared ACK^53^ bands at 1698, 1582 and 1427 cm^−1^ all ascribed to C=O stretching of the carboxylic groups, C=C of the aromatic groups and –OH bending.^60,61^ The stretching vibration of C–O–C bonds of esters, phenol, carboxylic and ethers^62,63^ are prominent at 1150–1079 cm^−1^, indicative that the produced carbon samples have abundance oxygen moieties on the surface of ACK samples.^53^ The weak peak at 2300 and 870–750 cm^−1^ are ascribed to the C–H sp^3^ stretching present in the lignin^53^ and out-of-plane bending vibration of C–H in the aromatic rings.^64^ Moreover, the peaks of aliphatic C–H stretching and stretching vibration of C–O–C bonds were decreased slightly with increasing IR and MPT, highlighting the dehydrating influence of KOH^65^ and intense microwave heating during the activation process.^66^ The prepared ACKs samples possess hydroxyl, carboxyl and aromatic functional groups on their surface, which offers promising route for extensive functionalization. These functional groups make the ACKs samples more hydrophilic in nature, in turn effectively aid dispersion of catalyst nanoparticles on the ACK surface. The hydrophobic nature of these functional groups from the prepared ACKs samples ensures the good metal-halide-support interaction.^67^

**Fig. 5:**
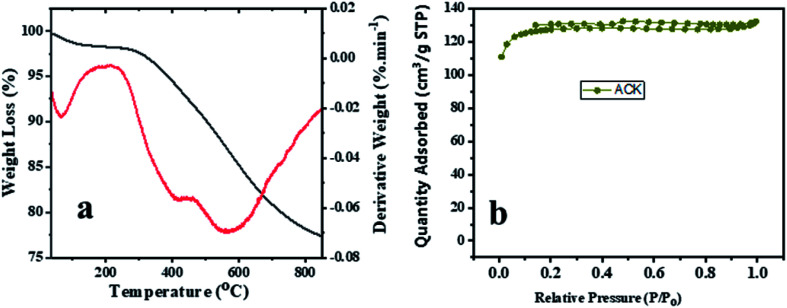
(a) Thermogravimetric and differential thermal analysis profiles and (b) nitrogen adsorption–desorption isotherm of ACK-2.24-16 sample.

The TGA and DTA analysis of ACK-2.24-16 sample is shown in Fig. 5a. The ACK sample was conducted in N_2_ atmosphere at temperature range between 35 to 850 °C and showed three-weight loss in Fig. 5. The first weight loss occurs between 30–154 °C with loss of 3%, which is ascribed to decomposition of water molecules adsorbed on the carbon surface.^68^ The second weight loss (8%) for the ACK happens between 154–480 °C, which is due to decomposition of hemicellulose, cellulose and lignin structure in form of volatile matters and inorganic matrix elimination.^69^ The last stage of weight loss occurs between 480 and 680 °C with weight losses from 12%, is attributed to decomposition of extra residual carbonaceous skeleton.

The N_2_ adsorption–desorption isotherms for the optimized ACK-2.24-16 is shown in Fig. 5b. The samples have a typical type IV isotherm, which indicates that they are mesoporous materials,^70^ with a contribution of micropores which correlates with the IN and MBN adsorption studies. Both samples also presented type H4 hysteresis loop in line with IUPAC nomenclature showing the presence of slit-shaped pores.^71^ The Brunauer–Emmett–Teller (BET) surface area calculated from the N_2_ sorption isotherms are 427.45 m^2^ g^−1^ for ACK-2.24-16 while the pore volume is 0.203 cm^3^ g^−1^. The prepared ACK-2.24-16 sample obtained in this study present considerable moderate BET surface area (427 m^2^ g^−1^) in comparison to other AC produced from Peanut shell (96 m^2^ g^−1^),^72^ Yellow mombin fruit stones (167 m^2^ g^−1^),^73^ coconut coir (205 m^2^ g^−1^)^74^ and coconut shell (478 m^2^ g^−1^).^75^ This further suggest that KOH activation of pinecone along with microwave pyrolysis approach is a good route to produce activated carbon as potential photocatalyst support.

Fig. S2† shows that the pH_PZC_ of ACK-2.24-16 is equal to 8.5. Invariably, for pH values lower than 8.5, ACK-2.24-26 sample will have positive charge on its surface and negative charge for pH values higher than pH_PZC_. It should be highlighted that the basic attributes of ACK-2.24-16 is ascribed to presence of carbonyl, pyrone and chromene groups^76^ present on the AC surface and is in agreement with FTIR result (Fig. 4c and d). The adsorption capacity of the cationic methylene blue in activated carbon increases with pH due to the basic surface groups *via* electrostatic interactions^77^ and form the basis for carrying out adsorption experiment at pH 12 (as stated above in adsorption studies). These basic functionalities on AC surface have also shown potential to enhance electrochemical activity in a carbonaceous material.^78^

### 3.2. Adsorption studies on prepared ACK samples

**Fig. 6:**
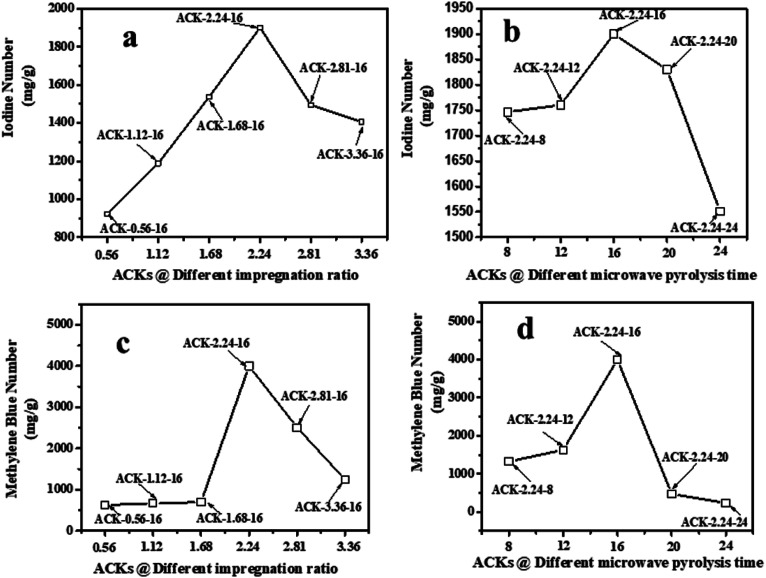
Iodine number and methylene blue number capacity of ACK samples at different (a and c) impregnation ratio and (b and d) microwave pyrolysis time.

The adsorption experiment studies *via* iodine number and methylene blue number capacity for the prepared ACK samples are further discussed in Fig. 6a–d. From Fig. 6a and b, the IR microwave time have more significance on the iodine number (IN) adsorption capacity compared to MPT. The IN adsorption is significantly reduced at lower IR (0.56–1.12) and MPT (8–12 min) with IN capacity range from 797.10 to 1188.06 mg g^−1^. Herein, an incomplete porous structure formation (from Fig. 2 and 3) with low active sites results from insufficient reaction between the carbonized material and activating agent (KOH). At low IR, more MPT is required for development of porous channel (micropores) to aid IN adsorption. Increasing IR from 1.68–2.24 and MPT to 16 min results into an increment of IN adsorption, as the optimum IN capacity of 1900 mg g^−1^ for ACK-2.24-16 as shown in Fig. 6a and b. The porous channels within the 3D hierarchical network structure is evident with ACK-2.24-16 sample from Fig. 2 for high IN capacity, as volatile matters that are detrimental to formation of this porous channel (micropores and mesopores) are been eliminated.^79^ Further increment in IR from 2.81–3.36 and MPT from 20–24 min after the optimum ACK sample results in decrease in IN adsorption in Fig. 6a and b. This reduction in IN capacity is attributed to destruction of porous channel (formation of macropores) (as seen in Fig. 2 and 3) and results to blockage of the porous channels.^80^

The methylene blue number (MBN) capacity for the ACK samples is shown in Fig. 6c and d, the effect of IR were more dominant on MBN adsorption capacity as compared to MPT. The MBN capacity is reduced at lower IR (0.56–1.68) and MPT (8–12 min) as depicted in Fig. 6c and d. However, an increment in IR to 2.24 and MPT at 16 min results into higher MBN adsorption capacity up to 4000 mg g^−1^. The optimum MBN capacity of 4000 mg g^−1^ was obtained for ACK-2.24-16 sample as shown in Fig. 6c and d. The porous channel for ACK-2.24-16 along with increase active sites are factors for higher MBN adsorption capacity. However, an increase above this condition results into decrease in MBN adsorption capacity for ACK. Herein, the increment in both factors above the optimum condition causes excessive dehydration and collapse of porous channels (macropores formation), invariably reduces the adsorption efficiency of ACK. The optimized ACK 2.24-16 sample IN and MBN capacity was further compared with other AC produced from agricultural biomass using microwave and conventional route with KOH chemical impregnation as presented in Table S1.† The higher IN and MBN adsorption capacity for ACK shows that pinecone is an efficient biomass source for good carbon production with well-developed porous structure. Sample ACK-2.24-16 with good porous channel (abundant micropores and mesopores, less of macropores), will offer enormous interspace for mass transport and pollutant adsorption.^54^ The developed 3D hierarchical network structure for ACK-2.24-16 sample with abundant micropores and mesopores, will also enhance charge carrier transport that results to exceptional electrochemical attributes^55^ for a supported catalyst as further discussed in the electrochemical studies for ACK samples.

### 3.3. Electrochemical properties of ACK samples

**Fig. 7:**
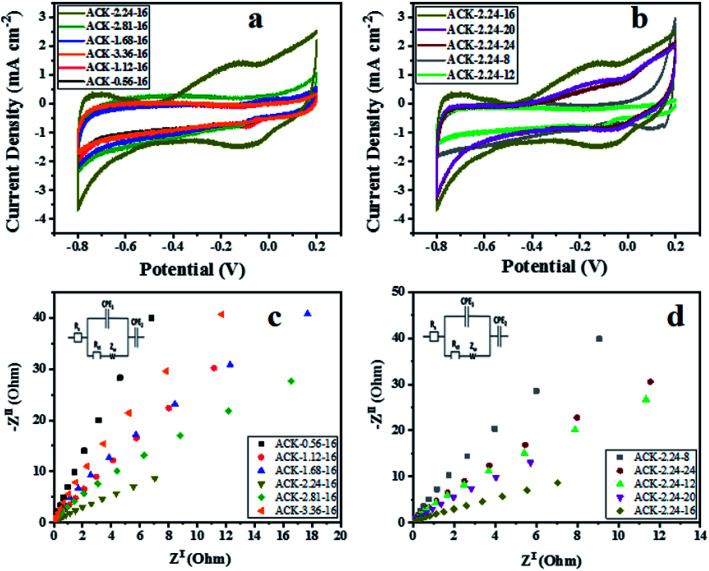
Cyclic voltammetry curves, electrochemical impedance spectroscopy of ACK samples at different (a and c) impregnation ratio and (b and d) different microwave times.

The electrochemical properties of ACKs were further evaluated in a three-electrode system with 6 M KOH as an aqueous electrolyte and the results are shown in Fig. 7a–d. The cyclic voltammetry (CV) curves of ACKs electrode at different IR and MPT (Fig. 7a and b) collected at scan rate of 50 mV s^−1^, exhibited a quasi-rectangular shape with bumps (caused by redox reactions). It observed from the CV curves, that the ACK-2.24-16 sample shows the largest current response demonstrating the best electrochemical properties among other samples (Fig. 7a and b). The high current response for ACK-2.24-16 is attributed to the 3D hierarchical network structure (with abundant micropore and mesopores) as indicated by the morphological and adsorption analysis. This effectively aid absorption of the electrolyte and minimize the diffusion resistance of the ion transport.^81,82^

Fig. 7c and d shows the Nyquist plots of ACKs electrodes in a frequency range from 10 kHz to 10 mHz. All the samples exhibit small semicircle in the high frequency and almost vertical-line feature in the low frequency, which are ascribed to charge transfer and Warburg resistance, respectively.^83^ Sample ACK-2.24-16 presents the smallest semicircle and shortest Warburg-type line among all the samples (Fig. 7c and d), which correlates with good interfacial charge transfer resistance (*R*_ct_) and faster ion transportation from the electrolyte to the inner mesopores.^84,85^ Overall, well developed 3D hierarchical porous structure of ACK-2.24-16 is key factor for good electrochemical properties for this material amongst the ACKs. ACK-2.24-16 sample with highest current response and least charge transfer resistance will be the optimized carbon sample to disperse Ag–AgBr catalyst, for efficient photocatalytic activity on the removal of tetracycline antibiotic under visible light irradiation.

### 3.4. Potential of ACK sample as catalyst support

The significant impact of the carbon sample (ACK-2.24-16) as catalyst support for Ag–AgBr was further evaluated to guarantee that the optimized carbon support applied throughout this study boost the performance of overall formed composite (Ag–AgBr-ACK) for tetracycline photodegradation.

**Fig. 8:**
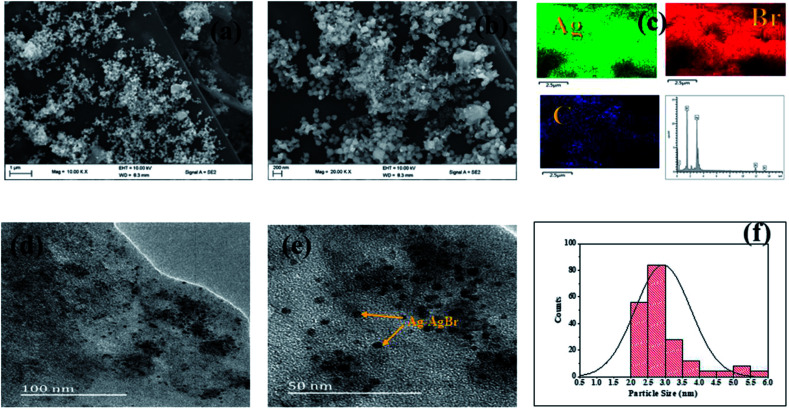
(a and b) SEM image; (c) elemental mapping and EDX spectrum of Ag–AgBr-ACK composite; (d and e) TEM images and particle size distribution (f) of Ag–AgBr-ACK composite.

Fig. 8a and b shows the SEM image of Ag–AgBr-ACK composite at low and high magnification. The 3D hierarchical network structure of ACK with abundant oxygenated functional groups significantly aid in the controlled dispersion and agglomeration prevention of Ag–AgBr nanoparticles, which will further favors a high catalytic activity. The EDX spectrum (Fig. 8c) was obtained to indicate the presence of Ag, Br and C elements in the Ag–AgBr-ACK sample. The TEM image for Ag–AgBr-ACK composite is spheres shaped like nanoparticles (Fig. 8d and e) and the PLAS particles with diameter around 3–5 nm (Fig. 8f) are uniformly distributed on the ACK surface.

**Fig. 9:**
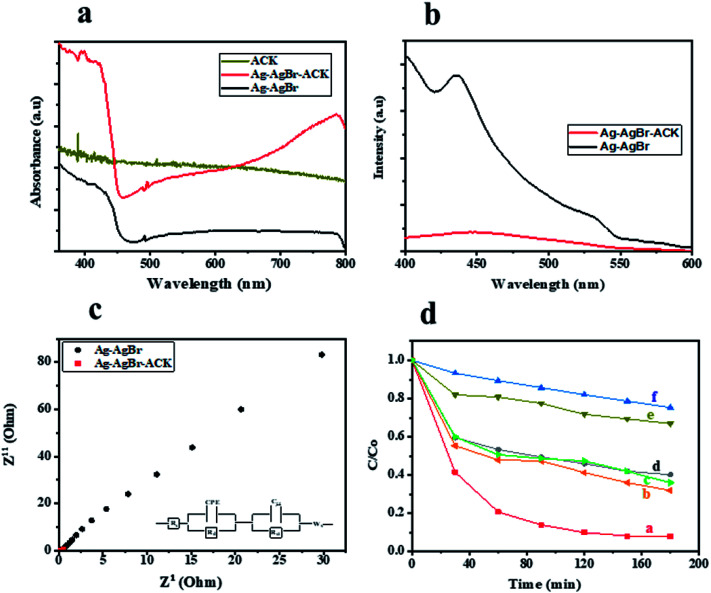
(a) UV-Vis DRS spectra of ACK, Ag–AgBr-ACK and Ag–AgBr; (b) PL emission spectra and (c) electrochemical impedance spectroscopy spectra of Ag–AgBr-ACK and Ag–AgBr; (d) relative concentration (*C*/*C*_0_) *versus* time plot of (a) Ag–AgBr-ACK, (b) Ag-ACK, (c) AgBr-ACK, (d) Ag–AgBr, (e) ACK and (f) photolysis.

UV-Vis DRS analysis was used to investigate the photoabsorption characteristics of Ag–AgBr-ACK, Ag–AgBr and ACK. From Fig. 9a, it can be observed that the ACK have absorption edge around 360–400 nm. Furthermore, the as-prepared composite Ag–AgBr-ACK showed enhanced absorption activity in the band region of 400–700 nm, attributed to the good dispersion of Ag–AgBr on the surface of ACK porous structure. The 3D hierarchical network structure from the carbon support (ACK) allows more light penetration, which further boost the light harvesting capacity of Ag–AgBr in the visible region than Ag–AgBr alone in Fig. 9a. The enhanced visible light absorption of prepared composite (Ag–AgBr-ACK) paves way for generation of more photogenerated charge carriers for high photocatalytic activity.^86^

Fig. S3† illustrates TGA results obtained for Ag–AgBr-ACK and ACK. The curve for the samples showed a mass loss of 2–3% when heated up to 150 °C because of adsorbed water loss. The samples shows significant mas loss from 150 to 400 °C, which is ascribed to the inorganic matrix elimination. The last weight loss due to decomposition of extra residual carbonaceous skeleton occurs between 400 and 600 °C. The thermal stability of the Ag–AgBr-ACK composite is lower than that of ACK, which indicates that the addition of Ag–AgBr nanoparticles slightly reduces the thermal stability of the composite material, which is similar with other report.^87^

The recombination of photogenerated charge carriers in all photocatalyst is a crucial issue, as a result photoluminescence spectroscopy (PL) analysis was evaluated on Ag–AgBr-ACK and Ag–AgBr sample. The PL analyses of both samples (Fig. 9b) indicated the main PL peak around 450 nm, attributed to the emission of the band gap transition. The peak intensity of PL spectra for Ag–AgBr-ACK is lower compared to Ag–AgBr, which suggests a lower recombination rate of photogenerated charge carriers.^88^ The reduced recombination rate emanates from 3D hierarchical network structure of ACK aiding fast interfacial charge carriers separation and migration of electron from Ag–AgBr conduction band,^89^ which aids high photocatalytic activity for the composite in pollutant removal.

The Ag–AgBr-ACK composite has a smaller arc radius diameter compared to Ag–AgBr as confirmed from the EIS Nyquist plot (Fig. 9c), indicative of charge carrier transfer efficiency. The ACK 3D hierarchical network structure in the composite (Ag–AgBr-ACK) significantly boost the efficient photoinduced electron transfer and interfacial charge separation. This fast separation of the photogenerated charge carriers by ACK is consistent with the PL spectra discussed above (Fig. 9b).

### 3.5. Visible-light photocatalytic activities

Tetracycline antibiotic (TC) was selected as a model PPCP to test the photocatalytic performance of prepared catalysts. TC is still very prevalent in the ecosystem,^90^ very difficult to be completely degraded under visible light by physical and biodegradation technique, as such the elimination of TC from the environment is a crucial issue.^91^ For comparison, the TC degradation under visible light irradiation by Ag–AgBr-ACK, Ag–AgBr, Ag-ACK, AgBr-ACK, ACK and photolysis were also evaluated (Fig. 9d). After 180 min irradiation by visible light, TC degradation was 92.08% in the presence of Ag–AgBr-ACK composite. With the prepared Ag–AgBr, Ag-ACK, AgBr-ACK and ACK, the percentage of degradations were 59.27, 68.2, 64.1 and 33.1% respectively. Higher photocatalytic activity of Ag–AgBr-ACK is attributed to 3D ACK hierarchical network structure aiding controlled growth of Ag–AgBr, which subsequently favours increase in visible light absorption and fast interfacial charge separation. The UV absorbance spectrum of TC at different irradiation times using Ag–AgBr-ACK composite are shown in Fig. S4a,† there is a sharp decrease in intensity without the emergence of new absorption peaks. Hence, no intermediates formed during degradation absorb at analytical wavelength and completely disappears within a period of 180 min showing the enhanced catalytic activity of Ag–AgBr-ACK composite. TC antibiotic in this study was mineralized into CO_2_ and H_2_O, which is proved by TOC analysis (Fig. S4b†). The Ag/AgBr and Ag/AgBr-ACK presented the 19% and 87.5% mineralization of TC, after 180 min under visible light irradiation. It also indicates that Ag/AgBr NPs decorated on the ACK 3D hierarchical porous structure enhances mineralization process 5 times fold. A comparison result of photocatalytic degradation of Ag/AgBr-ACK composite with other reported works using different support materials for Ag/AgBr photocatalyst is presented in Table S2.† The results shown here indicated that ACK-2.24-16 with 3D hierarchical network structure is a promising catalyst support material that can be explored in boosting metal semiconductor catalyst properties for environmental remediation.

## 4. Conclusion

In conclusion, activated carbon was prepared by two stage pyrolysis route in this study. The activated carbon were synthesized from waste biomass, through microwave pyrolysis of chemically activated pinecone by KOH. The pinecone biomass was utilised for the first time as green carbon source to create environmental friendly, highly efficient catalyst support material. The ACKs possesses 3D hierarchical network structure with abundant micropores and mesopores, have plentiful oxygenated functional groups on their surface along with good electrochemical properties making the ACKs a potential catalyst support material. The optimized 3D ACK hierarchical network structure had a high surface area of 427 m^2^ g^−1^. The 3D ACK 2.24-16 hierarchical structure material significantly boost the characteristic properties of Ag–AgBr in the prepared composite (Ag–AgBr-ACK) as a potential catalyst support. A higher photocatalytic activity of Ag–AgBr-ACK on degradation of tetracycline antibiotic under visible light is ascribed to 3D ACK hierarchical structure abating the controlled growth of Ag–AgBr with enhanced visible light absorption and fast interfacial charge separation. ACK shows superior performance as catalyst support for Ag–AgBr dispersion in pollutant removal compared to other support. Overall, this study offers an interesting perspective on boosting catalytic performance of catalyst for environmental remediation and promotes biomass as good precursor for activated carbon production as catalyst support.

## Conflicts of interest

The authors declare no conflict of interest.

## Supplementary Material

RA-010-C9RA10638C-s001
