# Supplementary material for: Three-dimensional hierarchical porous carbon structure derived from pinecone as a potential catalyst support in catalytic remediation of antibiotics
Source: RSC Adv. 2020 Feb 28;10(15):8717–28. doi: 10.1039/c9ra10638c (PMC9049983; doi:10.1039/c9ra10638c)
Supplement: RA-010-C9RA10638C-s001 [file RA-010-C9RA10638C-s001.pdf]

Electronic Supplementary Material (ESI) for RSC Advances.
This journal is © The Royal Society of Chemistry 2020

Three-Dimensional Hierarchical Porous Carbon Structure Derived from Pinecone as Potential Catalyst Support in Catalytic Remediation of Antibiotics

Sanni S.O., Viljoen, E.L, Ofomaja A.E**

Biosorption and Wastewater Treatment Research Laboratory, *Department of Chemistry, Faculty of Applied and Computer Sciences, Vaal University of Technology, P. Bag X021, Vanderbijlpark-1900, South Africa.*

**Corresponding author's address: E-mail aus_ofomaja@yahoo.com; augustineo@vut.ac.za
Phone: +27 745408161

Experimental section

The synthesis of Ag/AgBr on activated carbon (Ag-AgBr-ACK) was conducted through an existing synthetic approach [1]. Typically, 18 mL of ethylene glycol (EG, 99%, Acros) was poured into a round-bottom flask which was heated at 60 ℃ for 30 min. Polyvinylpyrrolidone (0.26g, PVP, M.W. 58000, K 29-32 Acros) and 480 mg of Hexadecyltrimethylammonium bromide (HTAB, Acros, 99%) were sequentially added to the solution, and then 0.03 g of ACK were further then added to the reaction mixture. After proper mixing to completely dissolve the PVP and HTAB, 3 ml EG solution containing 200 mg silver nitrate (Merck, 98%) was slowly added drop-wise to the stirred solution. The solution was maintained at 60 °C for an additional 30 min, and thereafter the stirred solution was then heated to a temperature of 140 °C. After reaching the desired temperature, the reaction was further stirred for 18 min. Thereafter the reaction mixture was cooled down to room temperature in the air. The resulting precipitate were separated by

centrifugation at 6000 rpm for 10 mins (REMI bench top centrifuge-R-8 D), washed thoroughly with ethanol, and dried in an oven for 12 hr. For comparison purpose, Ag-ACK, AgBr-ACK were prepared as described above in the dark without addition of HTAB and in the dark condition. While, Ag/AgBr was prepared the same route above in the absence of ACK. UV-vis diffuse reflectance (DRS) analysis of Ag/AgBr, Ag-AgBr-ACK and ACK carried out with an Ocean Optics high-resolution spectrometer (Maya 2000), using BaSO4 as the reference material. Photoluminescence (PL) spectra of Ag/AgBr-ACK and Ag/AgBr detected under FP-8600 Spectrofluorometer, Jasco at an excitation wavelength of 365 nm.

2.4 Catalytic activity test on tetracycline degradation

The prepared photocatalyst (Ag/AgBr-ACK, Ag-ACK, AgBr-ACK, Ag/AgBr and optimized ACK) were evaluated in tetracycline (TC) mediated system under visible light irradiation. The reaction was carried out under a 36 W white visible RGB LED light [2]. For all the samples, 0.3 g/L were added in 150 mL aqueous solution of TC (15 ppm). Prior to irradiation, the mixed solution was sonicated, mildly stirred in the reactor for 60 min in the dark to reach adsorption/desorption equilibrium. Then the LED light illuminated the mixed solution, the concentration of TC analyzed by a UV-visible spectrophotometer (T80 model, PG Instrument Limited, USA) at 376 nm every 30 min.

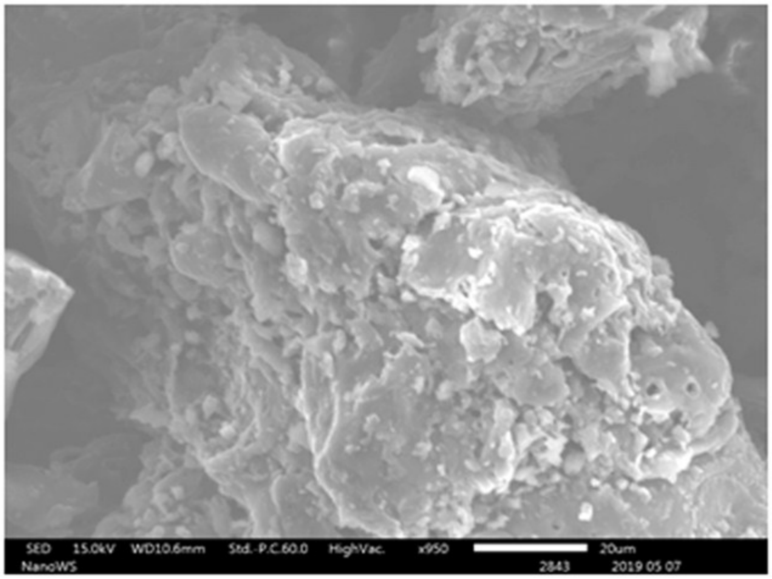

Fig. S1. SEM image of carbonized material.

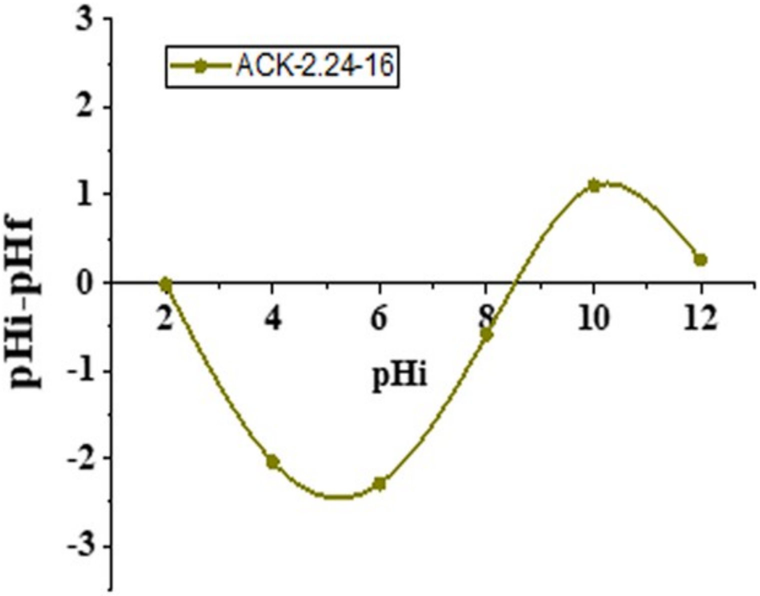

Fig. S2. pH-pzc of ACK-2.24-16 sample.

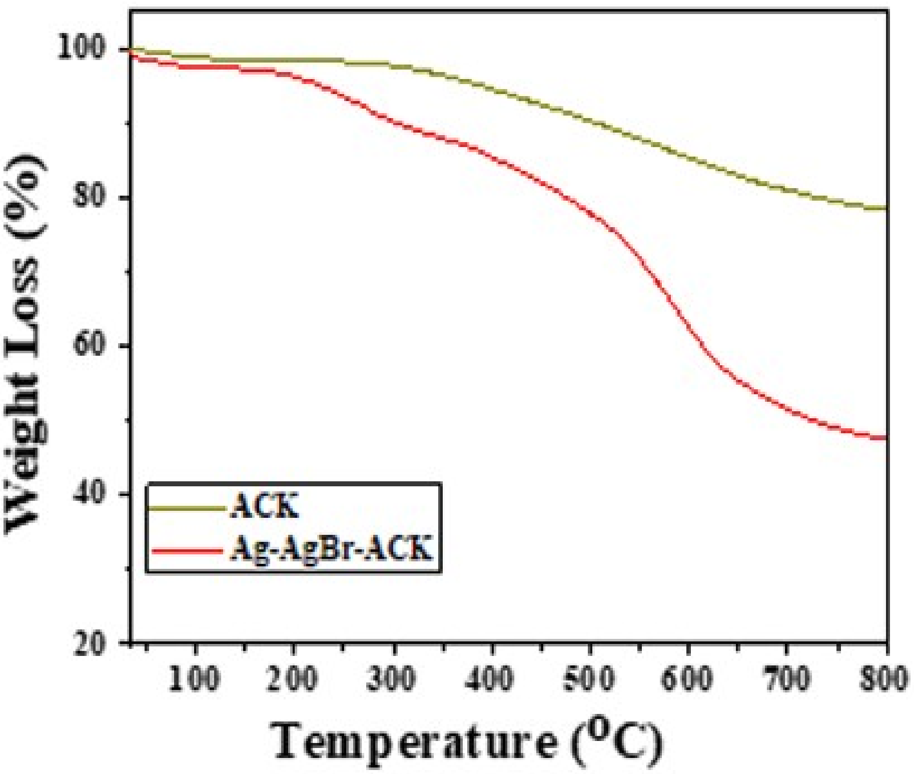

Fig. S3. TGA curves of ACK and Ag-AgBr-ACK

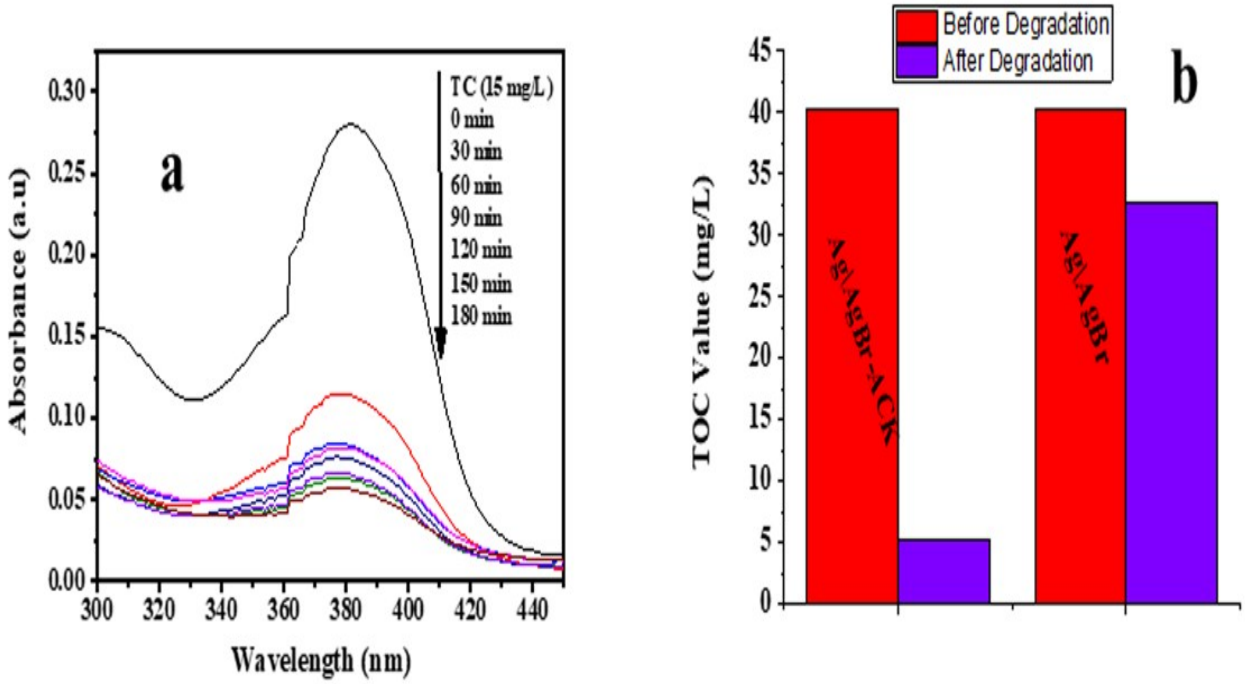

Fig. S4. (A) UV–Vis absorbance spectra of TC reduction in the presence of Ag-AgBr-ACK; (B) TOC reduction of TC using Ag/AgBr-ACK and Ag/AgBr catalyst.

Table S1 : Comparison of percentage Yield, Iodine number and methylene blue number adsorption capacity based on different biomass derived carbons.

Biomass Material	Heating Method	% Yield	Iodine Number (mg/g)	Methylene Blue Number (mg/g)	Reference
**Pinecone**	Microwave	53	1900	4000	**This Study**
Oil palm fiber	Microwave	32.09	n.m.	382.32	[3]
Oil palm residues	Microwave	73.78	n.m.	395.30	[4]
Cotton Stalk	Microwave	n.m.	285.71	294.12	[5]
Siris seed pods	Microwave	22.48	1760.74		[6]
Hevea seed coat	Conventional	n.m.	n.m.	22.27	[7]
Durian shell	Conventional	n.m.	n.m.	289.26	[8]

n.m.: not mentioned

Table S2 : Comparison of degradation efficiency for different support materials for Ag-AgBr.

Catalytic Material	Catalyst dosage (g/L)	Light Source	Pollutant, concentration (mg/L)	% Degradation, irradiation time (min)	Reference
Ag-AgBr-ACK	0.3	36 W Visible LED Light	Tetracycline, 15	92 after 180 min	**This study**
PNIPAM@AgBr/CSs	1	500 W Xe lamp	Tetracycline, 20	79 after 60 min	[9]
Ag@AgBr/SBA-15	0.3	500 W Xe lamp	Rhodamine B, 20	88 after 300 min	[10]
Ag–AgBr@hierarchical Hollow silica spheres	1	300 W halogen lamp	Methyl Orange, 10	97 after min	[11]
Ag/AgBr/$NiFe_2O_4$	1	10 W LED lamp	Rhodamine B, 10	89 after 60 min	[12]
Graphene Oxide Enwrapped Ag/AgBr	0.6	500 W xenon arc lamp	Methyl Orange, 15	85 after 40 min	[13]
Ag/AgBr–montmorillonite	1.4	100W tungsten filament Philips lamp	Methylene blue, 3.2	92 after 120 min	[14]

References

1. G. Chen, F. Li, Z. Huang, C.-Y. Guo, H. Qiao, X. Qiu, Z. Wang, W. Jiang and G. Yuan, *Catal. Commun.,* 2015, **59**, 140-144.
2. E. O. Oseghe and A. E. Ofomaja, *J. Photochem. Photobiol. A: Chem.,* 2018, **360**, 242-248.
3. K. Y. Foo and B. H. Hameed, *Biores. Technol.,* 2012, **103**, 398-404.
4. K. Foo and B. Hameed, *Desal.,*2011, **275**, 302-305.
5. H. Deng, G. Li, H. Yang, J. Tang and J. Tang, *Chem. Engineer. J.,* 2010, **163**, 373-381.
6. M. J. Ahmed and S. K. Theydan, *J. Analy. Appl. Pyroly.,* 2013, **99**, 101-109.
7. B. Hameed and F. Daud, *Chem. Engineer. J.,* 2008, **139**, 48-55.
8. T. C. Chandra, M. Mirna, Y. Sudaryanto and S. Ismadji, *Chem. Engineer. J.,* 2007, **127**, 121-129.
9. P. Huo, Z. Ye, H. Wang, Q. Guan and Y. Yan, *J. Alloys Comp.,* 2017, **696**, 701-710.
10. L. Hu, H. Yuan, L. Zou, F. Chen and X. Hu, *Appl. Surf. Sci.,* 2015, **355**, 706-715.
11. M. Shakeel, B. Li, M. Arif, G. Yasin, W. Rehman, A. U. Khan, S. Khan, A. Khan and J. Ali, *Appl. Catal. B: Environ.,* 2018, **227**, 433-445.
12. M. Ge, W. Liu, X.-R. Hu and Z.-L. Li, *J. Phys. Chem. Sol.,* 2017, **109**, 1-8.
13. M. Zhu, P. Chen and M. Liu, *ACS Nano,* 2011, **5**, 4529-4536.
14. S. Sohrabnezhad, A. Pourahmad and M. Razavi, *Appl. Phys. A.,* 2016, **122**, 822.
